# Evaluation and Management of Children with Congenital Heart Disease: From Age-Specific Diagnosis to Lifelong Care

**DOI:** 10.3390/children13081032

**Published:** 2026-08-03

**Authors:** Isabella Leo, Alfredo Marzano, Martina Avesani

**Affiliations:** 1 Cardiovascular Research Center, Magna Graecia University, 88100 Catanzaro, Italy; 2 Paediatric Cardiology Unit, Department of Woman’s and Child’s Health, University Hospital of Padua, 35128 Padua, Italy

Congenital heart disease (CHD) is the most common congenital malformation, affecting close to 9 per 1000 live births, and progressive advances in prenatal detection, surgical palliation, and perioperative care have transformed what was once an early-lethal condition into a chronic disease with a steadily expanding population of survivors reaching adolescence and adulthood [1,2]. This success has reshaped the central questions of the field. Where paediatric cardiology once measured itself largely by operative survival, attention has shifted toward the precision of early and subclinical diagnosis, the stratification and management of long-term risk, and the quality-of-life trajectories that increasingly determine how patients live with—rather than merely survive—their disease [3].

This Special Issue, “Evaluation and Management of Children with Congenital Heart Disease”, assembles contributions that trace this path from initial evaluation, through characterisation and therapy, to long-term outcome. Although CHD is the unifying thread, the collection deliberately reaches across paediatric cardiology—spanning screening and phenotyping, arrhythmia and device management, procedural safety, and psychosocial follow-up—because the contemporary care of patients with a structural heart defect is inseparable from these adjacent domains. Read together, the papers organise naturally around three questions: how to detect and phenotype disease with age-appropriate tools; how to characterise and manage arrhythmic and myocardial risk; and how to safeguard the whole child across a lifetime of care.

Accurate evaluation begins with tools calibrated to the developing heart. Two contributions address complementary facets of this challenge—one refining the reference standards of an established test, the other exploring the frontier of automated detection.

Miliaraki and Germanakis (Contribution 1) provide a narrative review of 16 studies reporting normal paediatric electrocardiographic values in the context of pre-participation sport screening. By synthesising evidence across contemporary reference datasets, the authors demonstrate that although developmental trends in ECG parameters are remarkably consistent, substantial heterogeneity persists, particularly for voltage-based criteria used to identify left ventricular hypertrophy. The review appropriately highlights how differences in population characteristics, ECG acquisition protocols, and measurement methodologies contribute to this variability, underscoring the need for standardised, contemporary, and population-representative reference values.

Where Contribution 1 refines human interpretation, Wazed et al. (Contribution 2) presented a proof-of-concept study, where a multimodal deep learning framework integrates heart sound signals with two-dimensional time–frequency representations to improve the detection of abnormal heart sounds in infants. The reported diagnostic performance is promising, suggesting that AI-assisted analysis of digital auscultation could enhance early screening for CHD in settings where advanced imaging or electrocardiography is not readily available.

Once disease is suspected, the goal is to focus on disease characterisation and risk stratification. Two reviews confront the heterogeneity that makes these tasks uniquely difficult in young patients.

Our group (Contribution 3) offers a comprehensive review of myocardial hypertrophy from prenatal life to young adulthood, illustrating why phenotype is only the starting point of a thorough diagnostic process. Identical ventricular wall thickening may reflect a transient, self-limiting process—as in the infant of a diabetic mother—or the earliest sign of a sarcomeric cardiomyopathy, a RASopathy, or a metabolic, mitochondrial, or storage disorder. Meanwhile, echocardiography of the first-line imaging modality, integrating cardiac magnetic resonance for tissue characterisation and molecular diagnostics for definitive aetiological assignment, is pivotal to achieving aetiologic precision [4,5].

Complementing this structural focus, Strangio et al. (Contribution 4) review arrhythmias across the paediatric age range, where mechanisms shared with adults coexist with entities specific to infancy, to congenital heart disease, and to inherited channelopathies. Advances in catheter ablation, electroanatomical mapping, and device technology have extended effective—and increasingly curative—rhythm management even to the smallest patients but often carry age-specific challenges.

For example, Suvorov and Ivanov (Contribution 5) reported a case of air trap within a pacemaker pocket in a child who required epicardial pacing after surgically acquired atrioventricular block complicating repair of a ventricular septal defect.

Finally, Schwartz et al. (Contribution 6) address what is arguably the most consequential and least measured dimension of paediatric cardiac care: neuropsychological and quality-of-life outcomes in survivors of in-hospital cardiac arrest, most of whom had complex CHD. While overall quality of life was comparable to that of healthy peers, children exposed to prolonged or repeated resuscitation—and high-risk subgroups such as survivors of extracorporeal support—showed lower functioning across physical, emotional, social, and school domains, alongside elevated indicators of emotional and behavioural burden. Taken together, these data highlight the need to consider psychological well-being and quality of life as equally relevant outcomes to survival when assessing the long-term impact of paediatric cardiac arrest and its treatment.

Taken together, these six contributions highlight the evolution of paediatric cardiology toward a more personalised, lifelong approach to children with congenital heart disease. Advances in diagnosis, artificial intelligence, imaging, genetics, and risk assessment are improving care, but important gaps remain, including the need for better age-specific reference data, prospective validation of new technologies, and more effective long-term follow-up strategies. Importantly, outcomes should extend beyond survival, incorporating neurodevelopment, psychological health, and quality of life. The future of paediatric cardiac care lies in a lifelong, patient-centred paradigm that extends beyond cardiac survival to preserve development, well-being, and quality of life.
